# Spatial and Single‐Cell Multi‐Omics Analyses of T‐Cell Subsets and Interactions in Melanoma Lymph Node Metastasis

**DOI:** 10.1111/exd.70376

**Published:** 2026-10-14

**Authors:** Ras Azira Ramli, Samuel Tan, Yibi Chen, Glen Boyle, Quan Nguyen

**Affiliations:** ^1^ Faculty of Medicine Universiti Sultan Zainal Abidin Kuala Terengganu Malaysia; ^2^ Institute for Molecular Bioscience The University of Queensland Brisbane Queensland Australia; ^3^ QIMR Berghofer Brisbane Queensland Australia; ^4^ Frazer Institute The University of Queensland Brisbane Queensland Australia

**Keywords:** melanoma, single‐cell transcriptomics, spatial multi‐omics, T‐cell heterogeneity, tumour‐draining lymph node

## Abstract

Melanoma is a highly immunogenic malignancy, yet responses to systemic therapy remain variable. The tumour‐draining lymph node (TDLN), including the sentinel lymph node (SLN) during early regional spread, is not only a site of metastatic dissemination but also an important setting for T‐cell priming and immune regulation. Conventional classification of nodes as tumour‐positive or tumour‐negative provides essential staging information but reveals little about their immune state. Single‐cell immune transcriptomics, T‐cell receptor sequencing and single‐cell epigenomics now allow T‐cell states, clonal dynamics and regulatory programmes to be examined in greater detail. Spatial transcriptomics, multiplex imaging, spatial proteomics and emerging spatial metabolomics add the anatomical context needed to determine where these cells and molecular programmes occur within the intact tissue architecture. In this review, we examine how these approaches are being utilised to study T‐cell heterogeneity, spatial organisation and immune interactions within melanoma TDLNs, together with the emerging contribution of artificial intelligence (AI)‐assisted pathology. We also critically evaluate how these technologies are applied, with attention to tissue requirements, spatial resolution, molecular coverage, computational demands, scalability to archival haematoxylin and eosin (H&E) imaging cohorts and careful interpretation of predicted or inferred spatial features. By combining biological profiling with methodological appraisal, we highlight how multi‐omics immune signatures may refine nodal disease assessment and guide future biomarker development, while noting that clinical translation will require standardised analytical pipelines and disease‐specific validation across independent melanoma cohorts.

## Graphical abstract

This figure illustrates how single‐cell and spatial multi‐omics can be applied to map T‐cell states and immune organisation in melanoma tumour‐draining lymph nodes (TDLNs). During regional immune remodelling and nodal metastasis, immune‐active and immune‐suppressed regions may coexist within the same tissue. Spatial profiling can map putative immune niches, including HEV‐associated immune‐active regions, tumour‐adjacent exhaustion‐prone niches, stromal barrier regions, metabolic stress hubs and preserved lymphoid areas. These spatial patterns may help refine nodal assessment, support biomarker discovery and inform future immunotherapy strategies.

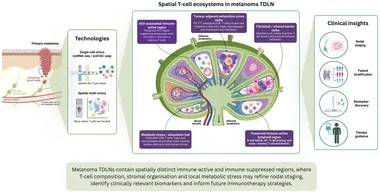

**Abbreviations**
AI: artificial intelligence
cDC1s: conventional type 1 dendritic cells
cDC2s: conventional type 2 dendritic cells
DNA: deoxyribonucleic acid
ECM: extracellular matrix
EV: extracellular vesicle
H&E: haematoxylin and eosin
HEVs: high endothelial venules
ICB: immune checkpoint blockade
ISH: in situ hybridisation
LN: lymph node
RNA: ribonucleic acid
scATAC‐seq: single‐cell assay for transposase‐accessible chromatin sequencing
scRNA‐seq: single‐cell RNA sequencing
SLN: sentinel lymph node
TCR: T‐cell receptor
TCR‐seq: T‐cell receptor sequencing
TDLN: tumour‐draining lymph node
Tex: exhausted T cells
Tfh: T follicular helper
TLS: tertiary lymphoid structures
Tpex: progenitor exhausted T cells
Tregs: regulatory T cells

## 1 Introduction

### 1.1 The Lymph Node as a Dual Metastatic and Immunological Site in Cancer

Lymph node (LN) metastasis represents a critical step in the progression of many human cancers, serving as both a marker of tumour dissemination and a reflection of evolving tumour‐immune interactions [1, 2]. While previously considered an anatomical junction for melanoma metastasis, the LN is now recognised as an active microenvironment in which malignant cells encounter immune, stromal, vascular and lymphatic components that may restrict or promote dissemination [3, 4]. Clinically, nodal involvement is a criterion for advanced‐stage disease and predicts increased risk of recurrence and mortality across a wide range of malignancies, including melanoma [1, 5].

However, anatomic definitions of nodal burden do not fully capture the immunological state of the node. Broadly, nodal invasion reflects both an increase in the tumour's metastatic potential and changes in anti‐tumour immunity within the lymph node. In melanoma, emerging single‐cell and spatial studies indicate that SLNs and TDLNs can contain tumour‐reactive immune cells, regulatory T cells, suppressive myeloid populations and exhausted T‐cell states organised within spatially distinct compartments [6, 7, 8]. Therefore, melanoma TDLNs are best evaluated as both metastatic and immunological organs. Emerging evidence also indicates that TDLNs may act as important sites for tumour‐specific T‐cell priming, maintenance and reinvigoration during immunotherapy, including the persistence of stem‐like or progenitor exhausted CD8^+^ T‐cell populations that may contribute to ongoing anti‐tumour responses [9, 10, 11].

### 1.2 Clinical Significance of the TDLN and SLN in Melanoma

Melanoma is a highly immunogenic malignancy [12]. Ultraviolet‐induced DNA damage contributes to a high mutational burden in melanoma, resulting in a diverse repertoire of neoantigens [13, 14]. However, invasive melanomas frequently develop mechanisms of immune escape, including upregulation of immune checkpoint molecules such as PD‐L1. This combination of high antigenicity and adaptive immune escape provides a strong biological rationale for the success of immune checkpoint blockade, particularly therapies targeting the PD‐1, PD‐L1, CTLA‐4 and LAG‐3 pathways [15, 16, 17]. Nevertheless, response rates remain heterogeneous, and it is unclear why many patients experience primary resistance or recurrence following an initial response. This highlights an urgent need for biomarkers that integrate tumour‐intrinsic features with the functional state of local and systemic anti‐tumour immunity.

In primary cutaneous melanoma, the sentinel lymph node (SLN) is identified through the injection of an intradermal radiotracer, which maps lymphatic drainage from the primary site and thereby indicates the most likely node for regional dissemination. SLN biopsy is routinely considered for tumour category T1b–T4 melanomas, and nodal positivity can directly qualify patients for adjuvant immune or targeted therapies [5, 18, 19, 20, 21]. As the most representative TDLN, the clinical relevance of the SLN extends beyond anatomic staging. It is also an immune organ exposed to tumour antigens, soluble mediators and extracellular vesicles (EVs) draining from the primary melanoma [22, 23, 24]. Even before overt metastasis is detectable, tumour‐derived signals may alter stromal organisation, dendritic cell function and regulatory immune populations [4, 23, 24].

Multi‐omics profiling of the TDLN may improve personalised estimates of recurrence risk and response to therapy. This rationale is particularly pertinent for Stage IIB/IIC with a negative SLN biopsy, where adjuvant immunotherapy is now approved [21, 25] but still requires careful risk–benefit selection, given the potential for immune‐related toxicities and the absence of definitive overall survival benefit [21]. High‐risk spatial features or checkpoint‐rich immune niches within the TDLN may identify subsets of node‐negative stage IIB/IIC patients with a greater likelihood of benefiting from adjuvant therapy.

### 1.3 Molecular Drivers and Bidirectional Tumour‐Node Crosstalk

Given its immunogenicity, melanoma must acquire mechanisms that allow it to evade immune clearance during both dermal invasion and regional nodal spread. Melanoma‐derived soluble mediators and extracellular vesicles can drain from the primary tumour through afferent lymphatics and contribute to pre‐metastatic niche formation in regional LNs [23, 24]. In particular, melanoma‐derived EVs have been shown to mediate lymphatic remodelling and impair tumour immunity in draining LNs [24]. Once melanoma cells colonise the lymph node, local metastatic deposits may further reinforce immune suppression through checkpoint‐regulated T‐cell dysfunction, regulatory immune populations and tumour–immune–stromal interactions within the node [6, 26, 27]. Conversely, the TDLN may also support systemic anti‐tumour immunity, as the proximity between draining tumour antigens and dense lymphoid architecture enables tumour‐specific T‐cell priming, maintenance and reinvigoration during immunotherapy [4, 10, 28]. Recent preprint evidence suggests that tumour‐experienced CD8^+^ T cells may recycle through lymphatic routes to draining LNs, further supporting the TDLN as an active immune reservoir [29]. Although this finding requires peer‐reviewed validation, it reinforces the importance of considering the TDLN as an active immune reservoir rather than a passive staging site.

As the TDLN becomes progressively tolerogenic, its capacity to sustain effective tumour‐specific CD8^+^ T‐cell responses may be compromised. Tumour‐induced immune dysfunction within the TDLN may favour suppressive or poorly functional lymphocyte states, reducing the quality of anti‐tumour immunity available to the host [11, 26]. In particular, checkpoint‐mediated regulation within TDLNs may restrain tumour‐specific T‐cell immunity [27]. In parallel, dysfunction of stem‐like or progenitor‐like T‐cell populations may impair the immune reservoir required for effective checkpoint‐mediated reinvigoration [9, 10, 11]. Thus, multi‐omics profiling is important for characterising how melanoma‐associated immune dysfunction is organised across nodal compartments, including tumour‐containing regions, checkpoint‐regulated T‐cell dysfunction and lymphatic remodelling [6, 24, 27, 30].

### 1.4 Resolving Intra‐Nodal Heterogeneity in the Melanoma TDLN

Despite the clinical importance of melanoma LN metastasis, a central biological question remains incompletely resolved: how does the TDLN transition from a physiologic site of immune surveillance and T‐cell priming into a tolerogenic pre‐metastatic or metastatic niche? This shift is unlikely to represent a uniform loss of immune activity throughout the node. Instead, melanoma‐associated immune remodelling is likely to occur through non‐uniform changes across nodal compartments, including checkpoint‐mediated T‐cell dysfunction, lymphatic remodelling, and metastasis‐associated immune‐cell states in melanoma SLNs [6, 24, 26, 27].

Although bulk transcriptomics can identify broad molecular changes associated with metastasis, the aggregation of signals across heterogeneous cell populations obscures cell‐type‐specific and spatially restricted effects. Flow cytometry and immunohistochemistry add important phenotypic information, but are constrained by predefined marker panels, limited multiplexing or loss of tissue‐wide molecular context. These approaches cannot fully determine where dysfunctional T‐cell states occur, which neighbouring cells they encounter, or whether immune‐active regions persist within the same node.

To address this gap, single‐cell and spatial multi‐omics provide complementary platforms for studying melanoma LN metastasis at higher resolution. Single‐cell approaches can characterise transcriptional states, clonal relationships and regulatory programmes, whereas spatial transcriptomics and spatial proteomics can localise these states within preserved tissue architecture. In this review, we synthesise how these technologies have shaped our understanding of T‐cell subsets, clonal dynamics and spatial interactions in melanoma TDLNs, while distinguishing evidence from tumour‐free TDLNs, tumour‐containing TDLNs, metastatic LNs, primary melanoma and non‐melanoma tumour settings where appropriate. The complementary roles of these approaches in resolving the melanoma TDLN are conceptually summarised in Figure 1.

**FIGURE 1:**
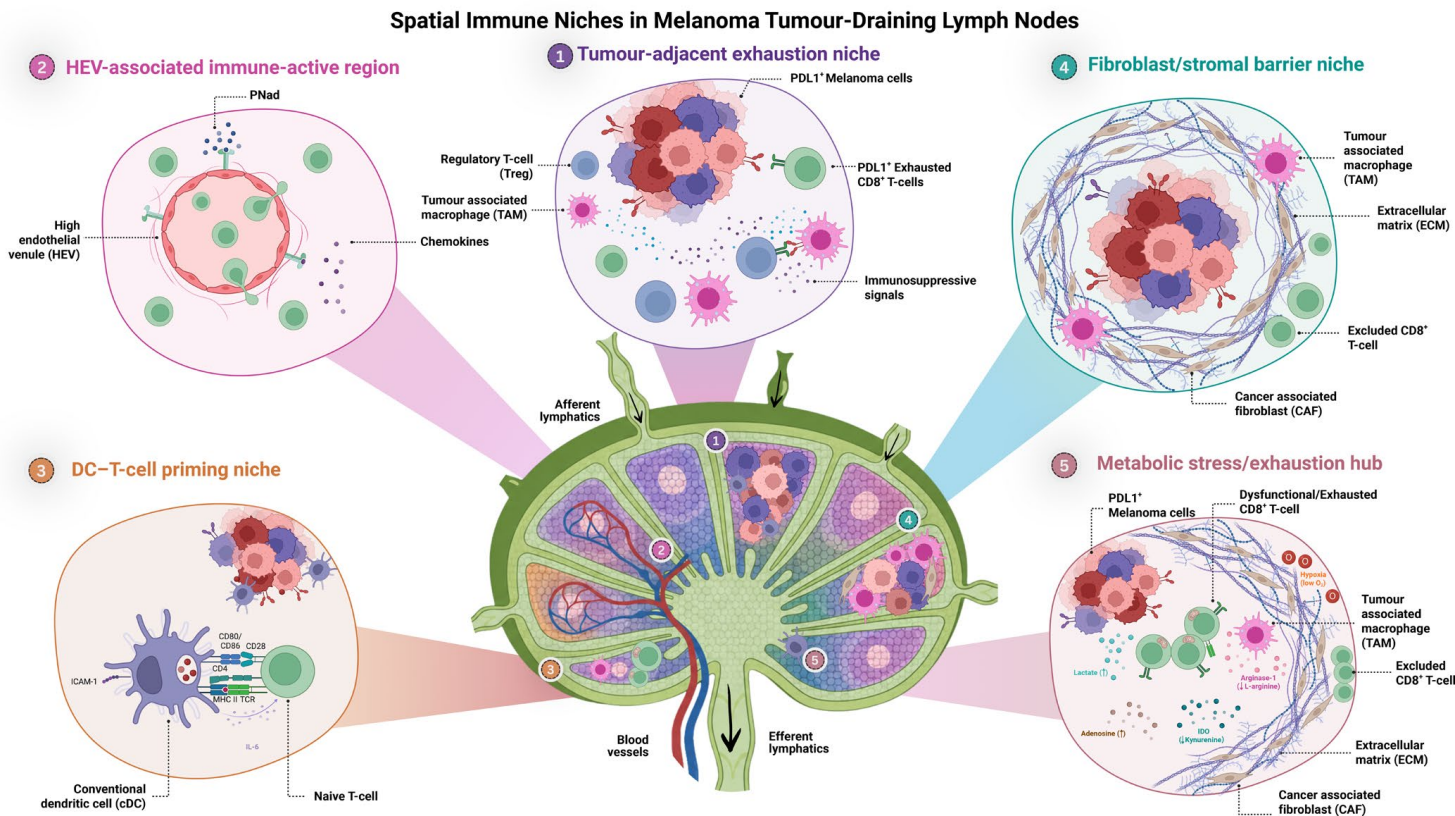
Spatial immune niches in melanoma tumour‐draining lymph nodes (TDLNs). A proposed framework suggests that melanoma TDLNs may contain multiple coexisting immune niches rather than a single uniform immune state. (1) In the tumour‐adjacent exhaustion niche, PD‐L1^+^ melanoma cells, Tregs, and tumour‐associated macrophages may localise near exhausted CD8^+^ T cells, potentially forming a suppressive checkpoint‐rich interface. (2) In the HEV‐associated immune‐active region, high endothelial venules may support lymphocyte entry and contribute to the maintenance of naïve or less differentiated T cells. (3) In the DC–T‐cell priming niche, conventional dendritic cells may support antigen presentation and T‐cell priming. (4) In the fibroblast/stromal barrier niche, CAFs, extracellular matrix remodelling and macrophages may restrict CD8^+^ T‐cell access to tumour areas. (5) In the metabolic stress/exhaustion hub, hypoxia, nutrient stress and suppressive metabolites may reinforce T‐cell dysfunction. Together, these niches illustrate how spatial organisation could shape immune surveillance, immune escape, and response to immunotherapy in melanoma TDLNs.

## 2 Application of Spatial and Single‐Cell Multi‐Omics to Investigate Melanoma Lymph Node Metastasis

A major challenge in studying melanoma LN metastasis is that the TDLN is a highly compartmentalised and heterogeneous organ. It contains diverse immune populations, stromal elements, lymphatic and vascular structures and frequently tumour cells that interact within a constantly adapting microenvironment. Because these components cannot be fully resolved by tissue‐level aggregation, single‐cell and spatial multi‐omics have become essential for identifying the cell states, molecular programmes and neighbourhood‐specific interactions that shape immune remodelling, immune evasion and metastatic progression within melanoma TDLNs. The key capabilities, biological applications and limitations of these technologies are compared in Table 1.

**TABLE 1:** Comparison of omics technologies in resolving the melanoma TDLN microenvironment.

Technology	Primary resolution	Established contribution in melanoma/melanoma LN studies	Relevance to melanoma TDLN biology	Current limitations
Bulk sequencing	Tissue‐level (averaged)	Identifies broad immune, tumour and stromal transcriptional programmes associated with melanoma progression, immune activation and LN metastasis [4, 23]	Useful for detecting overall immune activation, inflammation or suppression in melanoma LN tissue, but limited for resolving intra‐nodal heterogeneity	Averages signals across tumour, immune and stromal compartments; cannot resolve rare T‐cell states, clonal relationships, cellular neighbourhoods or spatial organisation
scRNA‐seq	Single cell	Resolves heterogeneous immune‐cell states in melanoma, including dysfunctional CD8^+^ T‐cell states, TCF7^+^ memory‐like or progenitor exhausted T‐cell populations, Tregs and myeloid subsets [31, 32]. Recent melanoma SLN scRNA‐seq has also identified immune‐cell signatures associated with nodal metastasis [6]	Allows identification of T‐cell exhaustion, regulatory programmes, dendritic‐cell dysfunction and macrophage states within melanoma SLN/TDLN samples	Loses spatial context; tissue dissociation may alter gene expression; fresh or high‐quality frozen tissue is often required; limited ability to infer direct cell–cell contact; transcriptomic states require protein‐level, spatial or functional validation
scTCR‐seq	Single cell/clonal	Tracks clonal expansion, persistence and replacement of tumour‐reactive T cells during immunotherapy. In melanoma, TCR profiling has shown that PD‐1 blockade can be associated with clonal replacement of tumour‐specific T cells [33]. TCR analysis has also been used to track tumour‐reactive clonotype dynamics associated with clinical response to TIL therapy in melanoma [34]	Useful for asking whether T‐cell clones in the TDLN are locally expanded, shared with primary tumour or metastatic sites, or associated with effector, progenitor exhausted or terminally exhausted states	Requires sufficient sequencing depth and matched tissue sampling; rare clonotypes may be missed; clonality does not automatically prove antigen specificity and requires antigen‐specific or functional validation [35]
scATAC‐seq	Single cell/chromatin accessibility	Defines regulatory programmes linked to T‐cell exhaustion and checkpoint response, including chromatin states associated with progenitor‐like versus terminally exhausted T cells [36, 37, 38]	Useful for distinguishing potentially reversible T‐cell dysfunction from more fixed exhaustion states in melanoma TDLN immune populations	Technically demanding; requires high‐quality nuclei or cells; not yet routinely applied to melanoma SLN/TDLN cohorts; interpretation often requires integration with scRNA‐seq and protein‐level validation
Spatial transcriptomics	Spot/sub‐cellular	Preserves tissue architecture while mapping transcriptional programmes. In melanoma, spatial profiling has revealed localised tumour–immune interactions, immunoediting and spatially organised immune suppression [39]. Spatially resolved cell–cell communication inference, including optimal‐transport‐based approaches such as COMMOT, can prioritise ligand–receptor relationships across tissue neighbourhoods, although these predictions require protein‐level, imaging or functional validation [40]	Allows localisation of effector T cells, exhausted T cells, Tregs, suppressive myeloid cells and tumour cells within preserved tissue architecture. Enables assessment of immune‐active and immune‐suppressed regions within sampled TDLNs	Trade‐off between resolution, gene coverage and tissue area; spot‐based platforms may contain multiple cells and have limited sensitivity for rare cell states; requires careful deconvolution and validation. Inferred cell–cell communication requires protein‐level, imaging or functional validation
Spatial proteomics/multiplex imaging	Single‐cell to subcellular protein‐level spatial resolution	In melanoma, multiplex imaging and spatial proteomic approaches have shown that immune‐cell spatial organisation, marker co‐localisation and tumour–immune neighbourhoods can inform response to immune checkpoint blockade [41, 42, 43]. High‐plex spatial protein profiling has also demonstrated the feasibility of mapping T‐cell and dendritic‐cell organisation across tumour and non‐tumour regions of melanoma SLNs [8]	Useful for simultaneous mapping of effector, regulatory and immune‐checkpoint markers, including CD8, FOXP3, PD‐1, PD‐L1 and CD163, within melanoma LN tissue	Usually limited to preselected marker panels; antibody validation is critical; lower discovery capacity than transcriptome‐wide methods, but stronger for protein‐level and spatial validation of transcriptomic findings
Spatial metabolomics	Spot/sub‐cellular	Still emerging in melanoma. Spatial immunometabolism provides a framework for mapping nutrient gradients, metabolic stress and immunosuppressive metabolites within tissue niches [44]	May help assess whether exhausted T cells in melanoma TDLNs localise to metabolically permissive or constrained regions	Limited validation in melanoma TDLNs; specialised equipment and analytical workflows are required; integration with transcriptomic, proteomic and spatial‐cell‐state data remains challenging
AI‐assisted histology‐to‐spatial prediction	Computational inference from H&E morphology	Deep‐learning methods suggest that histological image features can be used to infer spatial gene‐expression patterns from H&E‐stained tissue sections [45]. More recent AI‐based approaches further indicate that routine histopathology images may contain information predictive of spatially variable genes and tissue neighbourhood patterns, although current validation has mainly been outside melanoma TDLN cohorts [46]	As diagnostic H&E slides are widely available, this approach could support large‐scale screening of archival melanoma SLN cohorts for predicted immune signatures and spatial patterns, including exhausted T‐cell niches, myeloid‐rich suppressive regions and preserved lymphoid zones	Predictions are computational estimates rather than direct spatial measurements; melanoma‐specific and lymph‐node‐specific validation is required before biological or clinical interpretation

### 2.1 Single‐Cell Transcriptomics and TCR Profiling

Single‐cell RNA sequencing (scRNA‐seq) and single‐nucleus RNA sequencing have revolutionised the study of immune heterogeneity in cancer by enabling transcriptomic profiling at the cellular level. In melanoma, single‐cell studies show that the T‐cell compartment comprises multiple transcriptional states, including activated, proliferative, dysfunctional/exhausted and progenitor‐like CD8^+^ T‐cell populations [31, 32, 47]. Recent scRNA‐seq analysis of melanoma sentinel lymph nodes further identified metastasis‐associated immune signatures, including greater differentiation towards a TOX2^+^/PDCD1^+^/CTLA4^+^ exhausted CD4^+^ T‐cell subtype in melanoma‐positive nodes [6].

Nevertheless, transcriptomic profiling captures transcriptional state rather than the full phenotypic and functional identity of immune cells. Gene‐expression data provide a powerful view of cellular programmes, but mRNA abundance does not always correspond directly to surface‐protein expression or functional capacity [48, 49]. This distinction is particularly relevant for T cells, where lineage, differentiation and checkpoint markers may be more reliably assessed at the protein level, and where unsupervised transcriptomic clustering may reflect dominant activation, cytotoxicity or stress programmes rather than canonical functional subsets [50, 51, 52]. Therefore, in melanoma TDLNs, scRNA‐seq‐derived T‐cell annotations are most informative when interpreted within a multimodal framework that includes protein‐level, spatial and functional validation.

This multimodal perspective is particularly important in melanoma tumour‐draining lymph nodes (TDLNs), where transcriptionally similar T‐cell populations may differ in localisation, protein phenotype or functional role [50, 53]. For example, activated, proliferative and exhausted T‐cell populations may share overlapping transcriptional modules related to cytotoxicity, interferon signalling or chronic antigen exposure [31, 32, 50]. Integrating transcriptomic data with TCR profiling, spatial proteomics and multiplex imaging provides a means of distinguishing whether these states represent tumour‐associated immune responses, bystander activation or locally suppressive immune programmes [33, 34, 35, 41, 42].

The value of single‐cell transcriptomics is further strengthened when paired with T‐cell receptor sequencing (TCR‐seq). TCR profiling links cellular phenotype with clonality, enabling investigation of clonal expansion, persistence and tissue distribution of tumour‐reactive T‐cell clones across tumour and nodal compartments [33, 34, 35]. In melanoma, TCR profiling has been used to show clonal replacement of tumour‐specific T cells following PD‐1 blockade and to track tumour‐reactive clonotype dynamics associated with response to TIL therapy [33, 34]. When applied to matched primary tumour, tumour‐free TDLN and tumour‐containing nodal specimens, TCR‐seq allows clonal relationships between progenitor‐like or exhausted T‐cell populations in TDLNs and tumour‐associated T‐cell populations to be examined, including less differentiated reservoirs implicated in later effector responses [9, 10, 54].

Despite these strengths, TCR‐based analysis remains technically challenging. Detecting and accurately interpreting rare clonotypes across multiple tissue sites requires careful experimental design, adequate sampling and sufficient sequencing depth. Additionally, many protocols require fresh or high‐quality frozen tissue, which may limit translation into routine dermatopathology workflows. Nonetheless, the ability to connect T‐cell state, clonality and tissue compartment makes scRNA‐seq combined with TCR‐seq a powerful approach for studying melanoma TDLN biology.

Although scRNA‐seq and TCR‐seq can characterise transcriptional T‐cell states and clonal relationships, they do not show where these cells reside within the lymph node. Hence, spatial context is required to localise these populations within tissue architecture and to assess regulatory, stromal and vascular perturbations associated with TDLN immune dysfunction.

### 2.2 Spatial Transcriptomics and Spatial Proteomics

Spatial transcriptomics and spatial proteomics address the loss of anatomical context inherent to dissociated single‐cell approaches by measuring molecular features within intact tissue architecture. This is especially important in LN profiling, where immune function is shaped not only by which cells are present, but also by how they are positioned relative to tumour, stromal, vascular and lymphoid compartments. Platforms such as spot‐barcoded Visium, in situ hybridisation‐based Xenium and CosMx, multiplex immunofluorescence and imaging mass cytometry allow investigators to map immune‐cell positioning and define cellular neighbourhoods [55, 56, 57, 58, 59]. Spatial transcriptomics can also support inference of spatially resolved cell–cell communication that may influence T‐cell activation, suppression or exhaustion [40].

This spatial perspective is particularly relevant to the melanoma TDLN, where immune modulation is likely to be focal and heterogeneous rather than uniform across the node. Tumour‐derived extracellular vesicles and local metastatic deposits may remodel regional LN immune organisation [6, 24]. In melanoma TDLNs, immune‐suppressive changes have been observed in interfollicular regions and melanoma‐positive SLNs, supporting the idea that exhausted T cells, regulatory T cells and checkpoint‐associated programmes may be spatially restricted rather than evenly distributed across the node [6, 7]. Macrophage‐driven suppressive neighbourhoods may further contribute to local T‐cell dysfunction, as LN macrophage‐derived IL‐33 can activate ST2^+^ Tregs and suppress CD8^+^ T‐cell immunity in melanoma models [60]. This distinction matters because an LN with abundant CD8^+^ T cells is not necessarily immune‐active if those cells are excluded from tumour‐rich areas, positioned within suppressive myeloid neighbourhoods or located near inhibitory checkpoint‐expressing cells. Spatial profiling extends the analysis beyond cell subset abundance and allows the functional organisation of the melanoma TDLN to be examined.

Beyond defining tissue architecture, high‐resolution spatial mapping is also important for identifying melanoma‐relevant spatial biomarkers. In conventional histopathology, nodal assessment is largely based on the presence, size and distribution of metastatic deposits. However, in highly immune‐mediated tumours such as melanoma, anatomical tumour burden alone may not fully reflect the immune state of the node. Spatial biomarkers may include the proximity between effector T cells and tumour cells, which has been linked to melanoma immune organisation and immunotherapy response in spatial imaging studies [41, 42]. Other candidate features include Treg‐ or macrophage‐enriched suppressive regions within melanoma TDLNs [7, 8, 60], co‐localised PD‐1/PD‐L1‐rich immune neighbourhoods [42, 43] and the preservation or disruption of immune‐active regions around vascular or lymphoid structures [8, 61]. These features may provide a more functional readout of whether a melanoma TDLN remains immune‐active or has shifted towards a tolerised, immune‐suppressed state.

Spatial proteomics is particularly valuable in this context because many clinically relevant immune markers are protein‐based and contextualised by cellular localisation. Multiplex imaging approaches can simultaneously assess melanoma cells, CD8^+^ T cells, Tregs, macrophage‐associated markers, dendritic cell markers and checkpoint molecule expression within the same tissue section [8, 42, 43]. This makes it possible to examine whether dysfunctional or exhausted CD8^+^ T cells are dispersed throughout the node or concentrated near tumour deposits [7, 8], whether suppressive myeloid populations form local barriers to T‐cell access [60, 62], and whether preserved immune‐active regions remain within otherwise metastatic tissue [8, 61]. These interaction‐based findings cannot be reliably inferred from bulk sequencing and are only partially captured by dissociated single‐cell datasets.

Notably, spatial technologies have significant technical trade‐offs (Table 1). There is often a balance between tissue coverage, spatial resolution and the number of molecular features that can be measured [55, 56, 57, 58, 59]. Array‐based platforms provide broad transcriptomic coverage but at spot‐level resolution, where each capture area may contain multiple cells [55, 56, 57, 58, 63, 64]. In contrast, ISH‐based technologies achieve near single‐cell or subcellular resolution but measure a smaller and pre‐specified panel [56, 57, 58]. Tissue age, fixation method, section thickness, RNA integrity and antibody performance can also affect data quality, particularly for clinical LN specimens [65, 66]. These constraints are important when interpreting spatial data from complex tissues such as LNs, where immune, stromal, vascular and tumour compartments are closely intermixed.

Spatial transcriptomics and spatial proteomics are able to localise immune‐cell states within the melanoma TDLN and provide an essential complement to scRNA‐seq and TCR‐seq. However, spatial localisation alone does not fully explain why some T‐cell states remain reversible, whereas others become fixed in dysfunctional programmes. Addressing this requires deeper regulatory and metabolic information, including epigenetic accessibility and local metabolic stress.

### 2.3 Single‐Cell Epigenomics (scATAC‐Seq) and Spatial Metabolomics

scRNA‐seq and spatial transcriptomics predominantly capture transcriptional output and do not directly measure the epigenetic and metabolic processes that also accompany T‐cell exhaustion. Once CD8^+^ T cells progress towards terminal exhaustion, checkpoint blockade may not fully restore their function if the underlying chromatin structure has become fixed in a dysfunctional state [36, 37]. Exhaustion‐associated transcription factors such as TOX and TOX2 contribute to durable exhaustion programmes during chronic antigen stimulation [67]. This gap is partly addressed through single‐cell assay for transposase‐accessible chromatin sequencing (scATAC‐seq), which can identify regulatory elements and transcription factor motifs linked to activation, memory, regulatory function or exhaustion [38]. Although scATAC‐seq has provided important insight into chromatin accessibility in intratumoral T‐cell exhaustion, its direct application to melanoma SLN or TDLN cohorts remains limited. Future melanoma TDLN studies could use scATAC‐seq to determine whether Tpex‐like states remain epigenetically poised for reinvigoration or whether terminally exhausted T cells show more fixed regulatory programmes.

In parallel, spatial metabolomics and mass spectrometry imaging provide complementary information by mapping nutrient gradients, metabolic stress and immunosuppressive metabolites within intact tissue [44]. Although these approaches remain technically specialised, integrating epigenomic, transcriptomic and spatial metabolic data may clarify why some T‐cell states remain reversible while others become deeply dysfunctional within tumour‐containing melanoma TDLNs or metastatic LNs.

### 2.4 AI‐Assisted Pathology for Spatial Biomarker Discovery

Despite their breadth and resolution, spatial and single‐cell omics platforms remain costly and technically demanding, restricting their uptake in the melanoma clinic. By contrast, haematoxylin and eosin (H&E)‐stained tissue sections are routinely obtained for melanoma diagnosis and can readily be digitised to support computer vision approaches. AI‐driven histology‐based methods are thus emerging as useful complementary tools for spatial biomarker discovery. Deep‐learning histology‐to‐spatial prediction models have shown that histological features from H&E images can be used to infer spatial gene‐expression patterns, linking tissue morphology to underlying molecular states [45]. This could extend the translational potential of spatial analyses, especially in larger retrospective cohorts with melanoma‐specific and overall survival data.

There is some precedent for automated T‐cell quantification in primary melanoma [68, 69], but these image‐based approaches do not resolve T‐cell subtypes or spatial molecular programmes. Histology‐to‐spatial prediction pipelines are trained on paired histology and spatial transcriptomics data, allowing gene‐expression patterns to serve as molecular labels for predicting spatial molecular features from routine tissue images [45]. Recent methods, including sCellST, extend this concept by predicting single‐cell gene expression from H&E morphology, although validation in melanoma TDLNs is still lacking [70]. Related work such as STimage‐1K4M has expanded the scale of paired histology image–gene‐expression resources for spatial transcriptomics, supporting the development of models that learn associations between H&E morphology and spatial molecular features [71]. These approaches remain exploratory for melanoma TDLNs because lymph‐node‐specific and melanoma‐specific training datasets are still lacking. Similarly, Path2Space uses deep learning to infer spatial gene‐expression information from routine histopathology images. Although not yet evaluated in melanoma TDLNs, initial applications in breast cancer suggest that tissue morphology may contain information predictive of spatially variable genes and neighbourhood patterns from standard H&E slides [46].

These models could potentially be employed to screen archived TDLN specimens for spatially resolved immune signatures, such as T‐cell exhaustion, tumour‐associated macrophage enrichment, or preserved immune‐active regions in otherwise immune‐cold nodes. Importantly, the majority of melanoma LN specimens are stored as archival H&E slides without matching spatial‐omics data. Pending prospective validation, gene expression and cell type prediction models may permit re‐assessment of archived clinical and trial datasets and consequently examine associations between immune features and recurrence risk or response to systemic therapy.

At present, histology‐to‐spatial prediction in melanoma TDLNs remains exploratory. Deep‐learning models infer molecular features from image morphology rather than measuring them directly. Additionally, melanoma‐specific training and validation datasets are still limited. Future studies should develop these models using melanoma‐specific spatial omics datasets, ideally with longitudinal outcome data. Until such validation is available, AI‐assisted spatial prediction should be viewed as a hypothesis‐generating tool that complements, rather than replaces, direct spatial measurement. Ultimately, these technologies are most powerful when used together rather than in isolation.

### 2.5 Integrating Single‐Cell and Spatial Data

Recent melanoma studies have combined single‐cell and spatial approaches to investigate tumour–immune organisation, treatment resistance and melanoma cell‐state plasticity, including a TCF4‐associated resistance programme mapped in tissue context [72, 73]. Although this study was not designed specifically to define melanoma TDLN immune states, it demonstrates the value of integrating single‐cell and spatial information to link melanoma cell plasticity with immune resistance.

Single‐cell data characterise cellular states, whereas spatial data position those states within native tissue architecture. AI‐assisted pathology may then extend selected spatial features to digitised H&E data, enabling discovery and validation analyses within larger archival cohorts. These approaches provide a more integrated view of melanoma as dynamic immune ecosystems rather than as collections of isolated cell types.

Computational tools such as RCTD and cell2location can deconvolve spatial transcriptomic profiles using scRNA‐seq references, enabling localisation of highly resolved cell states within native tissue sections [63, 64]. In melanoma TDLNs, integrated analyses can address questions that isolated modalities cannot resolve alone. These include where progenitor‐like and terminally exhausted T cells reside, whether tumour‐reactive clones localise near tumour deposits or immune‐active niches, and which myeloid or stromal neighbourhoods are associated with T‐cell dysfunction.

Functional dendritic cell networks are also relevant because conventional dendritic cells (cDCs) play an important role in coordinating antigen presentation and T‐cell priming. cDC2 populations have been implicated in supporting anti‐tumour CD4^+^ T‐cell immunity, suggesting that preserved dendritic‐cell–T‐cell neighbourhoods within the TDLN may be important for sustaining productive immune responses [74]. Findings from other immunogenic tumour settings, such as the TONIC trial in metastatic triple‐negative breast cancer, further support the broader idea that pre‐existing and inducible immune states can shape response to PD‐1 blockade. However, these findings should be used only as cross‐cancer rationale rather than melanoma TDLN‐specific evidence [75].

Overall, integrated single‐cell and spatial approaches provide a framework for moving beyond simple cell abundance towards a more functional understanding of melanoma TDLN biology. In melanoma TDLN, the relevant issue is not simply which immune populations are present, but where they reside and which cells occupy the same neighbourhoods. This distinction is particularly important when assessing which region is likely to support T‐cell activation instead of favouring tolerance or exhaustion. Such analyses place metastatic involvement within its immune and anatomical context rather than treating lymph nodes only as a site of tumour deposition.

## 3 T‐Cell Subsets and Interactions in Melanoma TDLNs

### 3.1 T‐Cell Landscape in Tumour‐Free and Tumour‐Containing Lymph Nodes

In non‐pathological lymph nodes, T‐cell populations are spatially organised into specialised zones that support immune surveillance, antigen presentation and adaptive immune coordination. Naïve and central memory T cells circulate through T‐cell zones, T follicular helper cells interact with B‐cell follicles and regulatory populations restrain excessive immune activation. This spatial organisation facilitates efficient antigen encounter and productive immune priming [76, 77, 78].

In melanoma, the immune state of regional LNs may begin to change before overt nodal metastasis is detectable. Tumour‐derived soluble mediators and extracellular vesicles can drain from the primary tumour to regional LNs and contribute to pre‐metastatic niche formation, including lymphatic remodelling and impaired tumour immunity [23, 24]. However, human evidence directly comparing tumour‐free melanoma TDLNs with matched non‐tumour‐draining control LNs remains limited. Therefore, immune changes in melanoma‐negative SLNs should be interpreted cautiously and distinguished from changes observed in melanoma‐positive or overtly metastatic LNs. Recent human melanoma data suggest that MCSP^+^ metastasis founder cells may acquire immune‐evasive programmes during early SLN colonisation, reinforcing the distinction between early nodal remodelling and established metastatic disease [79].

Once melanoma metastasis is established in the LN, local tumour deposits can further reshape the immune microenvironment. Recent scRNA‐seq analysis of melanoma SLNs identified metastasis‐associated immune signatures, including greater differentiation towards a TOX2^+^/PDCD1^+^/CTLA4^+^ exhausted CD4^+^ T‐cell subtype in melanoma‐positive nodes [6]. Together, these findings support the view that melanoma TDLNs should not be treated as immunologically uniform tissues, but as nodal environments in which tumour‐free and tumour‐containing contexts may show distinct patterns of immune remodelling.

Although this review focuses on T‐cell subsets and interactions, B‐cell follicles, Tfh–B‐cell interactions and plasma‐cell programmes are also important components of nodal immune organisation and may influence anti‐tumour immunity, warranting further investigation in melanoma TDLNs. Emerging evidence from melanoma and other tumour settings suggests that Tfh cells and B‐cell‐rich lymphoid structures may contribute to anti‐tumour immunity and immunotherapy response, although their role within melanoma TDLNs remains incompletely defined.

### 3.2 T‐Cell Subsets Within LN Metastases

Once melanoma metastasis is established in the LN, the T‐cell compartment undergoes further remodelling. Earlier frameworks often described T cells as broadly active or suppressed; however, single‐cell studies in melanoma have revealed a more complex continuum of transcriptional states, including activated, dysfunctional, proliferative and progenitor‐like CD8^+^ T‐cell populations [31, 32]. In melanoma‐positive or metastatic LNs, this complexity is likely to reflect both anti‐tumour immune priming and local immune suppression driven by chronic antigen exposure, tumour‐derived mediators and checkpoint‐regulated T‐cell dysfunction [6, 23, 24, 27].

Exhausted CD8^+^ T (Tex) cells exist across a differentiation hierarchy that includes progenitor exhausted T cells (Tpex), proliferative dysfunctional cells, effector‐like dysfunctional cells and terminally exhausted cells [31, 32, 80]. Tpex are commonly associated with TCF1, encoded by TCF7, and may retain self‐renewal capacity, proliferative potential and responsiveness to checkpoint blockade [80]. In contrast, terminally exhausted CD8^+^ T cells typically exhibit reduced TCF1 expression and upregulate multiple inhibitory receptors, including PD‐1, TIM‐3/HAVCR2, LAG‐3 and TIGIT, together with exhaustion‐associated transcriptional regulators such as TOX [31, 32, 67]. These terminal states may also become epigenetically constrained, limiting their capacity for durable reinvigoration after PD‐1 blockade and underscoring the importance of chromatin accessibility profiling for interpreting T‐cell dysfunction [36, 37, 38].

The distinction between Tex and Tpex is particularly relevant to the melanoma TDLN. TDLNs may preserve less differentiated tumour‐specific T cells, including TCF1^+^ progenitor‐like populations that can proliferate following checkpoint inhibition and generate or sustain tumour‐infiltrating effector clones [9, 10]. This LN‐as‐reservoir model has been demonstrated in lung adenocarcinoma and supported by studies of regional LNs in other tumour settings, where metastatic nodal involvement can disrupt dynamic CD8^+^ T‐cell responses to immunotherapy [9, 81]. However, the extent to which this hierarchy is preserved or disrupted in melanoma TDLNs remains underexplored. Further work using matched tumour–node datasets that integrate scRNA‐seq, TCR‐seq, scATAC‐seq and spatial profiling is needed to resolve these relationships.

The CD4^+^ T‐cell compartment is also remodelled during melanoma LN metastasis. Although T follicular helper and Th1‐like cells may persist, regulatory T cells can contribute to local suppression through checkpoint‐associated programmes and impaired dendritic‐cell–T‐cell priming within TDLNs [27, 82, 83]. In a recent scRNA‐seq study of 15 melanoma SLNs, melanoma‐positive nodes showed greater differentiation towards a *TOX2* ^+^/*PDCD1* ^+^/*CTLA4* ^+^ exhausted CD4^+^ T‐cell subtype, although no comparisons were drawn between melanoma‐negative TDLNs and healthy controls [6]. Collectively, melanoma LN metastases are characterised by a reorganised immune landscape in which tumour‐reactive T cells, Tpex‐like reservoirs, terminal Tex cells and suppressive Tregs may coexist within the same nodal tissue.

Remodelling the melanoma TDLN is unlikely to result from a single process. Chronic exposure to melanoma‐derived soluble mediators and EVs may promote pre‐metastatic niche formation and lymphatic remodelling [23, 24], while changes in dendritic‐cell priming, macrophage programmes and stromal organisation may contribute additional layers of immune dysfunction [60, 82, 83]. Transcriptomic profiling alone cannot fully resolve T‐cell state in melanoma TDLNs, as clonal relationships, positional cell–cell interactions and chromatin accessibility also carry biological significance. Integration of scRNA‐seq, TCR‐seq, scATAC‐seq and spatial profiling would allow direct investigation of whether Tpex remains in immune‐active regions, where terminally exhausted T cells are and how suppressive Tregs are spatially organised within metastatic LNs.

### 3.3 Myeloid and Dendritic Cell Crosstalk With T Cells

T‐cell fate in melanoma TDLNs is strongly influenced by antigen‐presenting and myeloid cells. Dendritic‐cell subsets play central roles in tumour‐antigen presentation and T‐cell priming. In particular, cDC1s support cross‐presentation to CD8^+^ T cells and have been linked to effective anti‐tumour immunity and checkpoint response, whereas cDC2 populations have been implicated in CD4^+^ T‐cell priming and broader immune coordination, suggesting that multiple dendritic‐cell subsets may contribute to anti‐tumour immunity within melanoma TDLNs [74, 83]. However, in tumour settings, dendritic cells may also adopt regulatory or tolerogenic programmes that limit productive T‐cell activation and contribute to immune evasion [82, 83]. In metastatic melanoma, mature and migratory dendritic‐cell states have been linked to immune infiltration and response to anti‐PD‐1 checkpoint blockade, reinforcing the importance of antigen‐presenting‐cell programmes in shaping T‐cell immunity [84].

Suppressive myeloid states may further shape T‐cell dysfunction within tumour‐involved or metastatic nodal environments. Pan‐cancer and melanoma studies have identified macrophage programmes, including SPP1^+^ and TREM2^+^ states, that are associated with immune evasion and extracellular‐matrix remodelling [85, 86]. Macrophages can also alter T‐cell accessibility by limiting CD8^+^ T‐cell movement and access to tumour cells [62]. In TDLNs, macrophages may also regulate T‐cell function through LN‐specific suppressive mechanisms. Phagocytosis of tumour‐cell debris by medullary sinus macrophages can induce IL‐33 secretion, which activates ST2^+^ Tregs and suppresses CD8^+^ anti‐tumour immunity in melanoma models; in human melanoma SLNs, higher IL‐33 expression was associated with disease stage and poorer survival outcomes [60]. Together, these findings suggest that macrophages can act as local regulators of T‐cell exclusion, motility and function within suppressive tumour‐associated neighbourhoods [60, 62].

As a whole, these interactions suggest that checkpoint immunotherapy may be insufficient to restore T‐cell function in a subset of TDLNs if antigen presentation is impaired or suppressive myeloid niches remain intact. In this setting, therapeutic benefit may depend not only on releasing checkpoint inhibition but also on maintaining productive dendritic‐cell–T‐cell priming and reducing macrophage‐associated barriers to T‐cell access or function. Spatial multi‐omics is well suited to test this possibility because this approach can determine whether suppressive myeloid populations actually occupy the neighbourhoods in which they are most likely to influence T‐cell fate.

## 4 Spatial Organisation of T‐Cell Subsets and Interactions

### 4.1 Exhaustion Niches and Tumour Boundaries

A key rationale for applying spatial multi‐omics to melanoma TDLNs is that immune dysfunction is unlikely to be evenly distributed throughout the node. Instead, exhausted or suppressive immune states may be concentrated within specific nodal compartments, particularly near tumour deposits, checkpoint‐rich interfaces or myeloid‐enriched regions. High‐plex spatial protein profiling of melanoma SLNs has demonstrated the feasibility of mapping T‐cell and dendritic‐cell organisation across tumour and non‐tumour nodal regions, supporting further investigation of immune‐suppressive and immune‐priming compartments within melanoma TDLNs [8]. Such spatial approaches provide a framework for identifying local tumour–immune interactions that may not be captured by bulk sequencing or dissociated single‐cell analysis.

Spatial profiling also provides a way to examine how metastatic colonisation may alter organised lymphoid architecture and promote suppressive local neighbourhoods. In LNs, stromal and reticular‐cell networks maintain chemokine gradients, including CXCL13, that organise B‐cell follicles and support productive immune‐cell positioning [87]. During metastatic colonisation, these lymphoid structures may become distorted, and the local immune repertoire may shift towards tolerance and dysfunction, although the extent and spatial pattern of this remodelling in melanoma TDLNs remain incompletely defined. Digital spatial profiling of matched primary tumours and TDLNs in a distant‐metastasis versus disease‐free Stage III melanoma cohort indicated enrichment of PD‐L1, CTLA4 and FOXP3 in distant interfollicular areas of the TDLN, suggesting that recurrence in advanced melanoma may be associated with transcriptomic alterations even in melanoma‐negative regions of the node [7]. Macrophages may further contribute to suppressive TDLN neighbourhoods, as LN macrophage‐derived IL‐33 has been shown to activate ST2^+^ Tregs and suppress CD8^+^ T‐cell immunity in melanoma models, with IL‐33 expression in human melanoma SLNs associated with disease stage and survival [60].

Spatial multi‐omics preserves the physical relationship between T cells, melanoma cells and suppressive immune populations. Although scRNA‐seq can characterise exhausted T‐cell transcriptional states, tissue dissociation removes the spatial context needed to identify which neighbouring cells these T cells encounter. Multiplex imaging approaches, including AstroPath‐based spatial analysis, have shown that quantitative assessment of PD‐1, PD‐L1, CD8 and macrophage‐associated markers can inform melanoma immune organisation and response to PD‐1 blockade [42, 43].

Although melanoma TDLNs may contain tumour‐reactive T cells, their presence does not necessarily indicate effective anti‐tumour immunity, particularly when these cells coexist with Tregs, suppressive myeloid populations and checkpoint‐rich tumour–immune interfaces [7, 8, 42, 43, 60]. These spatially resolved studies highlight the need to interpret immune‐cell abundance alongside tissue localisation and local cell–cell interactions. Although spatial immune signatures have not yet been validated as routine clinical biomarkers in melanoma TDLNs, they provide a framework for future studies aiming to distinguish immune‐active from immune‐suppressed nodal states and refine risk stratification beyond anatomical tumour burden.

### 4.2 Focally Compromised Vascular and Lymphoid Architecture in the TDLN

Blood and lymphatic vessels are key support structures for T‐cell immunity in the melanoma TDLN. Physiologically, vascular and lymphatic structures coordinate LN immune surveillance by delivering tissue‐derived antigen and dendritic cells through afferent lymphatics, while specialised high endothelial venules (HEVs) support the continuous entry of circulating naïve and memory lymphocytes [78, 88]. In melanoma, tumour‐derived mediators and EVs can remodel lymphatic endothelium and impair anti‐tumour immunity in draining LNs, potentially altering leukocyte trafficking and local immune organisation [24, 89]. Recent melanoma work further links IFNγ‐dependent metabolic reprogramming with restraint of a pro‐metastatic lymphatic state, connecting lymphatic endothelial plasticity with anti‐tumour immune activity [90]. Notably, higher HEV density has been linked to increased stem‐like CD8^+^ T‐cell enrichment and a greater likelihood of response to PD‐1/CTLA‐4 combination therapy in metastatic melanoma [61]. These findings support further investigation of whether preserved HEV‐associated regions are associated with immune‐active compartments within melanoma TDLNs.

The term tertiary lymphoid structures (TLS) should be used cautiously in TDLNs, as LNs are already secondary lymphoid organs, whereas TLS typically describe ectopic lymphoid aggregates that develop in non‐lymphoid tissues [91]. In primary melanoma, mature TLS characterised by activated B‐cell follicles (CD23^+^), T‐cell zones and HEV‐associated architecture have been associated with improved survival and response to checkpoint blockade [91, 92, 93]. In TDLNs, the more relevant question is whether native lymphoid architecture is preserved, disrupted or remodelled into immune‐active versus immune‐suppressed microdomains. Spatial technologies make it possible to examine whether B‐cell follicles, HEV‐associated regions, dendritic cell–T‐cell neighbourhoods and tumour‐adjacent suppressive zones coexist within the same node.

This framing avoids treating metastatic LNs as a single immune state. Instead, melanoma TDLNs may be better understood as spatially heterogeneous nodal environments containing tumour‐adjacent exhaustion‐prone regions, macrophage‐rich suppressive neighbourhoods, disrupted lymphoid zones and preserved immune‐active compartments. Integrated single‐cell and spatial analyses extend the focus beyond immune‐cell composition by showing where immune populations are located, which neighbouring cells they encounter and how local tissue organisation may influence immune function. A proposed framework of these coexisting spatial immune niches is summarised in Figure 1.

## 5 Clinical Implications, Limitations and Future Perspectives

Biologically, single‐cell and spatial multi‐omics can move the field towards a more mechanistic and clinically informative understanding of melanoma LN metastasis. Logistically, these approaches may benefit from the routine use of SLN biopsy in clinically node‐negative melanoma, enabling discovery and validation across archival and prospective cohorts [18]. Pending validation across independent melanoma cohorts, multi‐omics TDLN profiling may improve patient‐specific estimates of recurrence risk and therapy response, particularly in patients with high‐risk node‐negative disease.

### 5.1 Beyond Anatomical Staging: Functional Immune Readouts

Current LN assessment is largely based on histological evaluation of anatomical tumour burden. While essential, this classification provides limited information about whether the nodal immune environment remains immune‐active or has shifted towards tolerance. Single‐cell and spatial technologies could complement conventional pathology by offering functional immune readouts that are not captured by tumour burden alone. These include the balance between Tpex and terminally exhausted T‐cell states, the proximity of effector T cells to Treg‐ or macrophage‐rich suppressive niches, the localisation of exhausted T cells at tumour boundaries, and the preservation of dendritic‐cell–T‐cell or HEV‐associated immune‐active regions.

This concept is supported by earlier molecular upstaging studies in melanoma, in which a subset of histopathology‐negative SLNs retained molecular evidence of occult disease associated with recurrence and survival [94, 95]. However, spatial immune biomarkers in melanoma TDLNs remain investigational. Multiplex proteomic, transcriptomic and AI‐assisted spatial approaches may identify immune‐active or immune‐suppressed nodal states, but melanoma‐specific validation is needed before these features can be used for routine clinical risk stratification.

### 5.2 Informing Therapy Response and Treatment Sequencing

The TDLN may provide a predictive window into tumour–immune dynamics because antigen presentation, T‐cell priming, immune suppression and metastatic colonisation can occur within the same nodal environment. In the neoadjuvant setting, preserving the nodal immune reservoir before surgical removal may be important for maximising systemic anti‐tumour responses. Spatial profiling may reveal whether a node contains preserved immune‐active regions that may respond to checkpoint blockade or, conversely, dense suppressive neighbourhoods that may require combination strategies targeting myeloid cells, Tregs, stromal remodelling or metabolic constraints.

Pilot data support the premise that TDLN immune dysfunction can be measured and may inform risk assessment in human melanoma. In Stage III melanoma, distant recurrence was associated with enrichment of Treg and checkpoint‐related signals in TDLN regions, including compartments without overt melanoma colonisation [7]. Similarly, scRNA‐seq profiling of melanoma SLNs identified metastasis‐associated immune remodelling, including greater differentiation towards a TOX2^+^/PDCD1^+^/CTLA4^+^ exhausted CD4^+^ T‐cell subtype in melanoma‐positive nodes [6]. Although larger longitudinal cohorts are required, these findings support the potential of TDLN profiling to delineate pre‐metastatic or metastatic immune remodelling and provide a complementary prognostic signal beyond primary tumour assessment.

### 5.3 Translational Limitations

Several barriers still limit the immediate clinical translation of spatial and single‐cell approaches. Most studies remain constrained by small cohort sizes, differences in tissue processing, batch effects and platform‐specific biases. Human data directly comparing melanoma‐negative TDLNs with matched non‐tumour‐draining or healthy control LNs are also limited, making it difficult to separate baseline nodal heterogeneity from tumour‐induced immune remodelling. Furthermore, definitions of functional cell states and spatial thresholds vary across studies, and analytical workflows are not always easily comparable. Collectively, these factors impair reproducibility and may challenge clinical implementation in a disease setting where timely management remains important [96].

Individual platforms also have distinct technical constraints. Single‐cell transcriptomics may underrepresent rare or adherent cell populations because of dissociation‐related biases, and transcriptional states do not always correspond directly to protein phenotype or functional capacity. Spatial transcriptomics platforms differ in gene coverage and spatial resolution, while array‐based methods may aggregate signals from multiple cells into a single capture spot. Multiplex imaging depends heavily on antibody‐panel design, protocol optimisation and image‐analysis pipelines. Inferred cell–cell communication from spatial transcriptomics should also be interpreted cautiously because spatial proximity and ligand–receptor expression do not prove direct functional signalling.

AI‐assisted pathology models have the potential to extend multi‐omics findings to larger clinical cohorts by inferring molecular or neighbourhood features from routine H&E slides. However, histology‐to‐spatial prediction tools require large, matched H&E and multi‐omics ground‐truth datasets, which have not yet been developed specifically for melanoma TDLNs [45, 46]. Future studies should prioritise matched tumour‐free and tumour‐containing nodal samples, protein‐level validation of transcriptomic T‐cell states, spatial validation of inferred cell–cell communication, and a deeper analysis of B‐cell follicles, Tfh interactions and plasma‐cell programmes within melanoma TDLNs. Until these datasets are available, preprint observations and computationally inferred features should be clearly distinguished from biomarkers that have been independently validated in melanoma TDLNs.

## 6 Conclusion

The melanoma TDLN is a dynamic immune environment in which tumour‐specific T‐cell priming and effector activity are counterbalanced by focal immunoregulation and progressive immune tolerance. Single‐cell technologies can characterise progenitor‐exhausted, effector, regulatory and terminally exhausted T‐cell states within melanoma TDLNs, together with antigen‐presenting cells and lymphatic or vascular structures that shape T‐cell differentiation. Spatial multi‐omics extends this information by mapping where these cells reside, which neighbouring cells they encounter and how local tissue architecture may influence immune function. These approaches are further complemented by clonotype analysis through scTCR‐seq, identification of accessible regulatory elements through scATAC‐seq, and translation of multi‐omics findings to archival H&E cohorts through AI‐assisted histopathology tools. Pending prospective validation of standardised analytical workflows in independent melanoma cohorts, TDLN profiling may improve risk stratification and estimates of therapy response, particularly for patients with high‐risk SLN biopsy‐negative primary melanoma. Figure 2 summarises the complementary information provided by these approaches.

**FIGURE 2:**
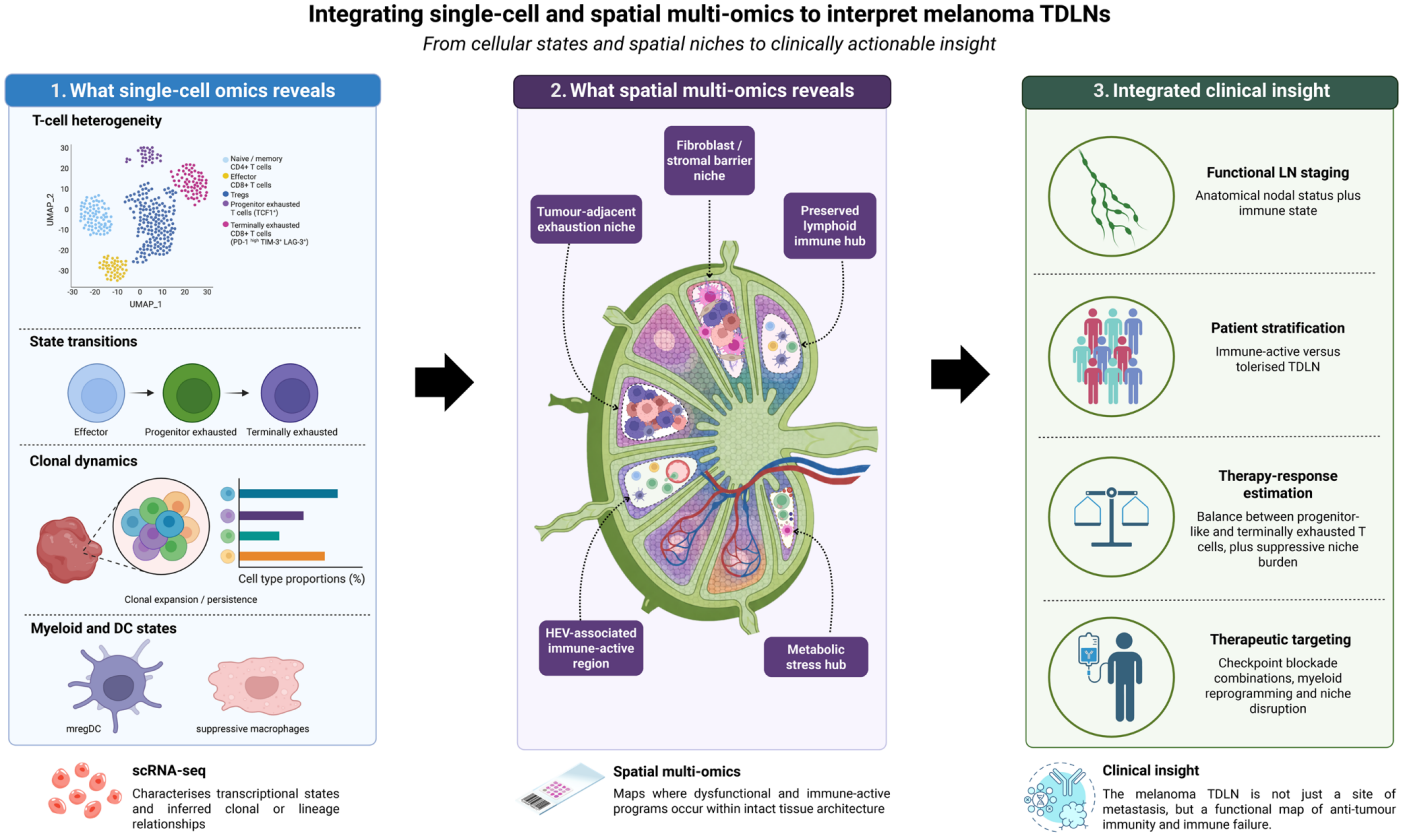
Integrating single‐cell and spatial multi‐omics to interpret melanoma TDLNs. The complementary roles of single‐cell and spatial multi‐omics in characterising melanoma tumour‐draining lymph nodes (TDLNs) are shown in three parts. (1) Single‐cell omics characterise T‐cell heterogeneity, transcriptional state transitions, clonal dynamics and myeloid or dendritic‐cell states. (2) Spatial multi‐omics maps where these immune states are located and identifies putative niches, including tumour‐adjacent exhaustion‐prone regions, stromal barriers, metabolic stress hubs, HEV‐associated immune‐active regions and preserved lymphoid immune hubs. (3) Integrated analysis may support functional interpretation of LN status, patient stratification, biomarker development and future therapeutic targeting. Together, these approaches highlight the melanoma TDLN as a spatially organised immune environment shaped by both anti‐tumour immunity and immune suppression, rather than solely as a site of metastasis.

## Author Contributions

Conceptualisation, Q.N. and R.A.R.; writing original draft preparation, R.A.R. and S.T.; writing review and editing, R.A.R., Q.N., Y.C., S.T. and G.B. All authors have read and agreed to the published version of the manuscript.

## Funding

This research was supported by National Health and Medical Research Council funding (NHMRC; Grant nos. 2001514 and GNT2008928). R.A.R. was funded by the Malaysian Ministry of Higher Education (MOHE).

## Conflicts of Interest

The authors declare no conflicts of interest.

## Data Availability

The authors have nothing to report.
